# Proviral integrations and expression of endogenous Avian leucosis virus during long term selection for high and low body weight in two chicken lines

**DOI:** 10.1186/1742-4690-6-68

**Published:** 2009-07-15

**Authors:** Sojeong Ka, Susanne Kerje, Lina Bornold, Ulrika Liljegren, Paul B Siegel, Leif Andersson, Finn Hallböök

**Affiliations:** 1 Department of Neuroscience, Uppsala University, Uppsala, Sweden; 2 Department of Medical Sciences, Uppsala University, Uppsala, Sweden; 3 Department of Animal and Poultry Sciences, Virginia Polytechnic Institute and State University, Blacksburg, USA; 4 Department of Animal Breeding and Genetics, Swedish University of Agricultural Sciences, Uppsala, Sweden; 5 Department of Medical Biochemistry and Microbiology, Uppsala University, Uppsala, Sweden

## Abstract

**Background:**

Long-term selection (> 45 generations) for low or high juvenile body weight from a common founder population of White Plymouth Rock chickens has generated two extremely divergent lines, the LWS and HWS lines. In addition to a > 9-fold difference between lines for the selected trait, large behavioural and metabolic differences between the two lines evolved during the course of the selection. We recently compared gene expression in brain tissue from birds representing these lines using a global cDNA array analysis and the results showed multiple but small expression differences in protein coding genes. The main differentially expressed transcripts were endogenous retroviral sequences identified as avian leucosis virus subgroup-E (ALVE).

**Results:**

In this work we confirm the differential ALVE expression and analysed expression and number of proviral integrations in the two parental lines as well as in F_9 _individuals from an advanced intercross of the lines. Correlation analysis between expression, proviral integrations and body weight showed that high ALVE levels in the LWS line were inherited and that more ALVE integrations were detected in LWS than HWS birds.

**Conclusion:**

We conclude that only a few of the integrations contribute to the high expression levels seen in the LWS line and that high ALVE expression was significantly correlated with lower body weights for the females but not males. The conserved correlation between high expression and low body weight in females after 9 generations of intercrosses, indicated that ALVE loci conferring high expression directly affects growth or are very closely linked to loci regulating growth.

## Background

Selection during more than 45 generations for low or high body weight from a common founder population of crosses among seven lines of White Plymouth Rock chickens has generated two extremely divergent lines; the low (LWS) and high weight selection (HWS) lines. The average body weight of individuals from each line differs by more than 9-times at 56 days, the age of selection. Numerous behavioural, metabolic, immunological, and endocrine differences between lines have evolved during the course of the selection experiment [1-4]. Among the obvious correlated responses to the selection for body weight were differences in feeding behaviour and food consumption. While HWS chickens are hyperphagic compulsive eaters and accumulate fat, LWS chickens are lean with low appetite. Some LWS individuals are anorexic even when fed *ad libitum *with 2 to 20% not surviving the first weeks post hatch because they never start to eat [5]. HWS chicks are put on a food restriction programme at 56 days to avoid health issues associated with obesity. A neural involvement in the development of the phenotypes was implied by results after electrolytic lesions of the hypothalamus [6]. We recently compared gene expression in brain tissue using a global cDNA array analysis with the purpose to reveal over-all expression differences between the HWS and LWS lines that may be causally related to their extremely different phenotypes. The results showed that the long-term selection has produced minor but multiple expression differences in protein coding genes. Genes that regulate neuronal development and plasticity such as regulators of actin filament polymerization and genes involved in lipid metabolism were over-represented among differentially expressed genes [7].

The most differentially expressed transcripts were sequences with similarities to endogenous retroviral sequences (ERVs) that were identified as avian leucosis virus subgroup-E (ALVE). Brain tissue of LWS individuals contained higher levels of transcripts encoding ALVE than that of HWS individuals. These results attracted our interest because the occurrence and frequency of ALVE proviral integrations in different chicken breeds have been shown to be associated with altered physiology [8], disease resistance [9] and reproduction efficiency [10]. The ALVE integrations are transmitted in a Mendelian fashion [11] and ALVE proviral integration frequency can change in response to selection for specific traits [12-15]. These data suggest that differences in ALVE integration between the LWS and HWS lines indicated by the large difference in expression may be related to the establishment of the extreme phenotypes of these selected lines.

Periodic sampling of the selected lines and the establishment of an advanced intercross line allowed us to test if there was a link between the observed differential ALVE transcript levels and body weights. Moreover, we were able to determine if the different ALVE expression was transmitted by inheritance or by congenital infection. The extent of proviral integrations and their relation to levels of ALVE expression were also analysed. The results show that high ALVE expression among F_9 _birds was significantly correlated with low body weight for the females but not for males. The conserved correlation between high expression and low body weight after 9 generations of intercrosses, indicated that ALVE loci conferring high expression are genetically linked to or constitute in part the loci for a low body weight of the pullets.

## Materials and methods

### Animals and tissues

Lines LWS and HWS were developed from a common founder population of crosses among seven inbred lines of White Plymouth Rocks, a breed used for egg production and broiler breeding. The selected lines have been maintained as closed populations by continuous selection for low or high body weight at 56 days of age for more than 45 generations. The average LWS and HWS chicks weigh 0.2 kg and 1.8 kg respectively at selection age. Descriptions of the selection programme and correlated responses of these lines are provided elsewhere [5,16]. All individuals sampled were from breeders of the same age, hatched on the same day, and provided feed and water *ad libitum*. Experimental procedures were approved by the Virginia Tech Institutional Animal Care and Use Committee. The founder lines as well as subsequent intercrosses were maintained at Virginia Polytechnic Institute and State University, Blacksburg, Virginia. The two lines have been kept in an identical and constant local environment during the course of selection. For example, each selected generation of the parental lines is hatched annually the first Tuesday in March and dietary formulation has remained constant throughout.

**Figure 1:**
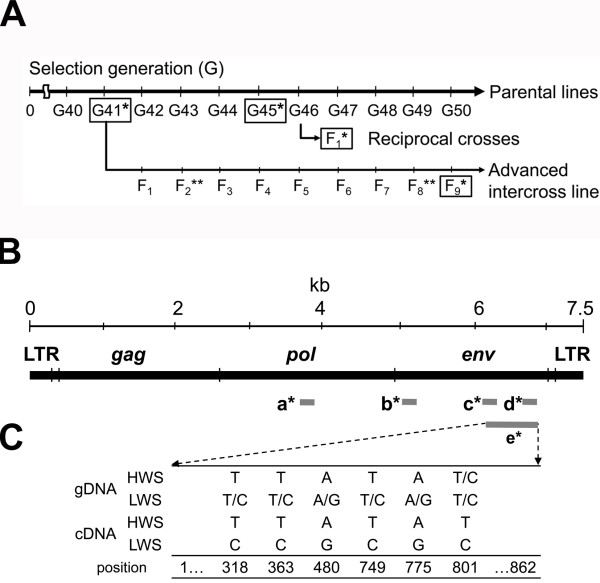
**Schematic ALV genome with PCR amplicons and SNPs**. **A**. Schematic time-line with parental generations and crosses. Generations in boxes were used for analyses in this study. Parental line generation (G) G41* and G45* were used to examine number of ALVE integrations. Expression studies were performed in the brain and peripheral tissues of G45* birds. F_1_* birds of the reciprocal crosses were utilized to test inheritance of ALVE genes. Eighty-two F_9_* birds that form the advanced intercross were utilized for the correlation studies. QTL analyses have been performed with F_2_** and F_8_** birds in the advanced intercross line [16,53]. **B**. Black bar represents a complete ALVE proviral genome. Grey bars indicate PCR primers and amplicons. **C**. Six SNPs between HWS and LWS lines were found in the 862 bp PCR fragment e* from both genomic DNA and cDNA. a*: pol197F/pol269R. b*: Val_envF/Val_envR. c*: env277F/env353R. d*: qPCR_envF/qPCR_envR. e*: an amplicon from a primer pair chENV232fwd/chENV1046rev. See table 1.

HWS and LWS chickens from generation 45 (G45, schematic outline of the generations Fig. 1A) were used for the cDNA array experiments and quantitative reverse transcription polymerase chain reaction (qRT-PCR) validation in peripheral as well as the brain tissues. Five or six males and five females from each line were sampled at hatch and at 56 days of age. Liver, pectoral muscle, adipose tissue and the brain region containing diencephalon, mesencephalon, pons, and medulla, were dissected on the day of hatch and at 56 days after hatch, immediately frozen in liquid nitrogen and stored at -70°C until used.

Reciprocal cross F_1 _chickens from G46 of the parental lines were used to test inheritance of ALVE expression. The intercross population between HWS and LWS chickens was produced with the main purpose to identify genes explaining the large difference in body weight and growth between the parental lines [16]. This intercross was initiated from G41 of the parental lines (see Fig. 1A). Eight HWS males were mated to 22 LWS females and 8 LWS males were mated to 19 HWS females to generate the F_1 _generation. The number of animals in F_9 _from the advanced intercross was 43 males and 43 females. Body weights at 56 days were recorded for all individuals. Livers were dissected for total RNA and genomic DNA preparation. Finally, 42 males and 38 females were used to measure relative mRNA amount of expressed ALVE with qRT-PCR.

Genomic DNA was used to analyse proviral integration number from HWS and LWS lines in both G41 and G45, 10 White Leghorn (WL) and 10 Red Jungle Fowl (RJF). The WL line (Line 13) originated from a Scandinavian selection and crossbreeding experiment [17] and was maintained at the Swedish University of Agricultural Sciences at a population size of 30 males and 30 females. The RJF birds originated from Thailand and were obtained from the Götala research station, Skara, Sweden. Information about the Line13 and RJF is published [18-20].

### Genomic DNA isolation

Genomic DNA from the parental lines and F_1 _chickens were isolated from blood following standard genomic DNA isolation method [21]. DNA from F_9 _chickens was isolated from liver using automated nucleic acid purification using GeneMole (Mole Genetics, Oslo, Norway) according to the manufacturer's guide.

### Total RNA isolation and cDNA synthesis

Each sample was homogenized into powder in presence of liquid nitrogen, followed by total RNA extraction with Trizol (Invitrogen Corporation, Carlsbad, CA, USA), and the quality of the total RNA was checked with the Agilent 2100 bioanalyser (Agilent Technologies, Santa Clara, CA, USA). One μg of total RNA was treated with RNase-free DNase (Promega Corporation, Madison, WI, USA) and used for cDNA synthesis with TaqMan Reverse Transcriptase reagents (Applied Biosystems, Foster City, CA, USA.) in a final volume of 50 μl containing 1 × TaqMan RT buffer, 2.5 μM random hexamers, 500 μM of each dNTP, 5.5 mM MgCl_2_, 20 U RNase inhibitor, and 62.5 U Multiscribe RTase. Samples were incubated for 10 min at 25°C, 30 min at 48°C, and 5 min at 95°C. The cDNA samples were stored at -20°C for storage.

### Tumour Viral locus B (TVB) genotyping

Genomic DNA samples of 10 HWS and 10 LWS birds (G41) were tested for genotyping of *TVB *alleles. A polymerase chain reaction-restriction fragment length polymorphism (PCR-RFLP) assay was performed following published procedures [22]. *TVB *genotypes were identified in 19 chickens, but the procedures failed to define a genotype for one LWS chicken.

### Cloning and sequencing of env fragments from cDNA and genomic DNA

Primers to amplify part of the *env *gene were designed in non-variable regions of the proviral *env *gene after aligning a number of sequences from GenBank. A primer pair, chENV232fwd and chENV1046rev, were used to amplify an 862 bp fragment from genomic DNA as well as cDNA as templates. Genomic DNA from 47 HWS and LWS individuals (G41) was used to amplify and sequence the 862 bp *env *fragment. cDNA samples of one male and one female representing the G45 parental lines were pooled and used for sequencing. Furthermore, cDNA from 14 F_9 _chickens were sequenced. The PCR was performed in a total volume of 10 μl containing about 50 ng genomic DNA or cDNA, 1× PCR Buffer (Qiagen, Valencia, CA, USA), 2× Q solution (Qiagen), 1.5 mM MgCl_2 _(Qiagen), 200 μM dNTP, 2 pmol of each primer and 0.5 U HotStarTaq Polymerase (Qiagen). Thermocycling started with 10 min at 94°C, followed by touchdown PCR cycling with denaturation 30 sec at 94°C, annealing 30 sec at 65°C and decreasing 1°C per cycle to 52°C and extension 1 min at 72°C. Thirty five cycles were then performed with 30 sec at 94°C, 30 sec at 52°C and 1 min at 72°C and the program ended with 5 min at 72°C. PCR products were separated in a 1% agarose gel and fragments excised and purified using QIAquick Gel Extraction Kit (Qiagen). PCR products generated from genomic DNA of parental lines and the expressed *env *fragments of F_9 _chickens sequenced directly using the PCR primers to obtain a representative sequence. PCR fragments from cDNA of parental lines were all cloned into pCR/GW/TOPO vector using TOPO TA cloning kit (Invitrogen) prior to sequencing with the T7 and M13R universal primers. Sequences were controlled, aligned and compared using the Sequencher 3.1.1 program (Gene Codes Corporation, Ann Arbor, MI, USA).

### Relative quantitative Reverse Transcriptase-PCR (qRT-PCR)

Two-step qRT-PCR was performed with the SYBR Green I Assay in combination with either ABI PRISM 7700 Sequence Detection System (Applied Biosystems), or MyiQ real-time PCR detection system (Bio-Rad Laboratories, Hercules, CA, USA) with iScript one-step RT-PCR kit with SYBR Green. One μl of the cDNA, derived from 20 ng of total RNA, was used as template in a 25 μl reaction mixture. PCR reactions were carried out in duplicates with activation of the polymerase for 10 min at 95°C and 40 cycles of two PCR steps, 95°C for 15 sec and 60°C for 60 sec. One-step qRT-PCR was used for analysis of *env *transcript levels in peripheral tissues of G45 and F_9 _chickens. Twenty ng of total RNA was added in 25 μl of the reaction mixture and then incubated for 10 min at 50°C for cDNA synthesis, for 5 min at 95°C for RTase inactivation and 35 cycles of two steps with 10 sec at 95°C and 30 sec at 60°C to amplify target transcripts. Primers used in all quantitative PCR (see Fig. 1B) were designed with Primer Express 1.5 software (Applied Biosystems) and are listed in table 1. A primer pair for quantitative PCR experiments, qPCR_envF and qPCR_envR, was designed within 862 bp of the PCR product described above. Chicken *β*-actin (GeneBank accession No. NM_205518) and glyceraldehyde-3-phosphate dehydrogenase (GAPDH, GeneBank accession No. NM_204305) were used as references. Each sample was assigned a CT (threshold cycle) value corresponding to the PCR cycle at which fluorescent emission, detected real time, reached a threshold above baseline. PCR products were separated in agarose gel to confirm that the products had the expected size. Collected data were normalized against the reference gene Ct values. Subsequently, relative mRNA expression levels of the test genes were determined in comparison with calibrators; for example, average expression levels of 0 day-old HWS males or shared subjects over the PCR plates. To examine whether the expression levels in HWS and LWS chickens were significantly different, one-way ANOVA together with Newman-Keuls post-hoc test in GraphPad Prism 3.03 (GraphPad Software, San Diego, California, USA) was utilized.

**Table 1:** List of the genes and primer pairs used for qPCR and qRT-PCR experiments

Primer names forward/revers	Amplicon in figure 1A	Forward	Reverse
Beta-actinF/Beta-actinR	-	AGGTCATCACCATTGGCAATG	CCCAAGAAAGATGGCTGGAA
GAPDHF/GAPDHR	-	GGGAAGCTTACTGGAATGGCT	GGCAGGTCAGGTCAACAACA
POMCF/POMCR	-	GCTACGGCGGCTTCATGA	CGATGGCGTTTTTGAACAGAG
PMCHF/PMCHR	-	CGAAATGGAGACGGAACTGAA	CATCCAAGAAGCTTTCCTCAATCT
Val_envF/Val_envR	b*	ACCCGGACATCACCCAAAG	AGTCAGAAATGCCTGCAAAAAGA
chENV232fwd/chENV1046rev	e*	ACGGATTTCTGCCTCTCTACACA	TTCCTTGCCATGCGCGATCCC
qPCR_envF/qPCR_envR	d*	GAAACTACCTTGTGTGCTGTCG	CGGATGTTGTGGAAAAACGA
env277F/env353R	c*	CCCAAAATCTGTAGCCATATGC	TACGGTGGTGACAGCGGATAGG
pol197F/pol269R	a*	TGCTTGTCTCCCCAGGGTAT	GGTGACTAAGAAAGATGAGGCGA

### Analysis of proviral integration in genomic DNA

The extent of proviral integration of ALVE was estimated by measuring the *env *proviral gene with qPCR in genomic DNA. The qPCR was performed as the qRT-PCR but with genomic DNA as template. Exactly 20 ng of the genomic DNA was analysed with primers qPCR_envF and qPCR_envR using a protocol with activation of the polymerase for 10 min at 95°C and 40 cycles of two PCR steps, for 15 sec at 95°C and for 60 sec at 60°C. Primers for chicken pro-opiomelanocortin (POMC, GeneBank accession NM_001031098) and pre-melanin-concentrating hormone (PMCH, GeneBank accession NW_001471513) were included in each of PCR plates as representatives for single-copy genes. All *env *Ct values were then normalized to the average of the POMC and PMCH Ct values and the relative *env *copy numbers were adjusted to the standard curve to get the *env *integration copy-number per haploid genome.

A plasmid (3679 bp) that contained the 862 bp *env *PCR product in pCR/GW/TOPO vector was used to make a standard curve. The plasmid was diluted serially in 2-fold, ranging from 0.02 ng to 0.16 pg per reaction volume in 8 dilutions, then qPCR was run together with qPCR_env primers and Ct values recorded. The number of the env-plasmids in each reaction was calculated. *n*; plasmid length (bp), *M*; average molecular weight of a base pair (650 g/mol), *N*_A _is Avogadro's constant, *m*; mass of the DNA.

Copy number of env plasmid = *m*/((*n *× *M*)/*N*_A_)

A standard curve was plotted using the plasmid number and the corresponding Ct values (2^-Ct^). A linear relationship was examined (y = 10^11^*x, R^2 ^= 0.9927). The number of haploid chicken genomes in 20 ng was also calculated using the chicken genome size *n *= 1.05 × 10^9 ^bp. There are 17650 haploid genome copies per 20 ng genomic DNA. The *env *gene integration number per genome for each individual was calculated using (10^11^*2^-Ct^)/17650.

## Results

### High ALVE expression in the LWS line

The differential expression of ALV-related sequences between lines LWS and HWS (G45) was found using a cDNA microarray analysis [7]. Brain tissue from both hatchlings and 56 day-old individuals of both sexes were analysed, and among the differentially expressed transcripts, at least 10 endogenous retrovirus-related transcripts were differentially expressed (p < 0.001) with high levels in the LWS line (Table 2, [23,24]). BLAST-search results using the array sequences revealed similarities to endogenous ALVE retrovirus elements. The fold difference between HWS and LWS lines varied from 2 to > 30-fold (Table 2).

**Table 2:** Differentially expressed virus-related sequence from cDNA microarray analysis

Probe ID	GeneBank ID	Gene annotation from the best hit/Domain	Fold difference of array expression (LWS/HWS)				Nucleotide BLAST		
				
			0 d male	0 d female	56 d male	56 d female	EST length	Hit length (hit/total)	Similarity (%)
RJA064A11.ab1	CN220264	ALV ev-21 and its integration site	30.8	20.6	23.8	21.5	377	305/2734	97.7
RDA-81	NA	ALV ADOL-7501, proviral sequence	20.0	12.9	10.4	11.2	210	207/7612	96.2
RJA002E06	CN216922	ALV strain ev-3/Avian gp85	18.4	13.6	14.6	14.4	757	757/5842	99.1
WLA044E07.ab1	CN223892	ALV strain ev-3, complete genome	8.3	5.7	6.8	6.2	588	409/5842	100
WLA070B07.ab1	CN230959	ALV strain ev-3, complete genome	6.9	4.9	7.8	6.9	368	153/5842	100
VeFi2.66.C3*	CN221614	Myeloblastosis-assoc. virus genes/Avian gp85	2.0	1.9	2.0	1.9	2567	2120/7704	92.3
WLA097G09.ab1	CN234473	ALV (strain RAV 7) 3' noncoding region	2.5	2.3	-.	2.1	326	274/358	94.5
WLA043C12.ab1	CN222802	ALV strain ev-3, complete genome	1.6	-	1.6	1.6	454	451/5842	96.3
WLA019C03.ab1	CN220591	ALV strain ev-6 envelope polyprotein	-	4.3	4.8	4.8	685	151/2720	96.7
RDA-69	NA	ALV strain ev-1, complete genome	-	-	-	2.5	185	170/7525	98.2

The gene annotation and BLAST result was collected from [23,24]. NA, GeneBank ID is not available. VeFi2.66.C3* had the best hit in Myeloblastosis-associated virus genes, however, BLAST result with protein sequences from SwissProt and TrEMBL showed the best hit on env protein of ALV.

Twenty-three virus-related sequences were arbitrarily selected from the array transcript list: nine ALV-related sequences, five other avian retrovirus-related sequences, (including Rous sarcoma virus transcription enhancer factor II, *env *gene of Rous sarcoma virus and gag/pol polyprotein of avian myeloblastosis virus), and nine retrovirus-related sequences from other species. Only the ALVE-related sequences were differentially expressed (data not shown).

**Figure 2:**
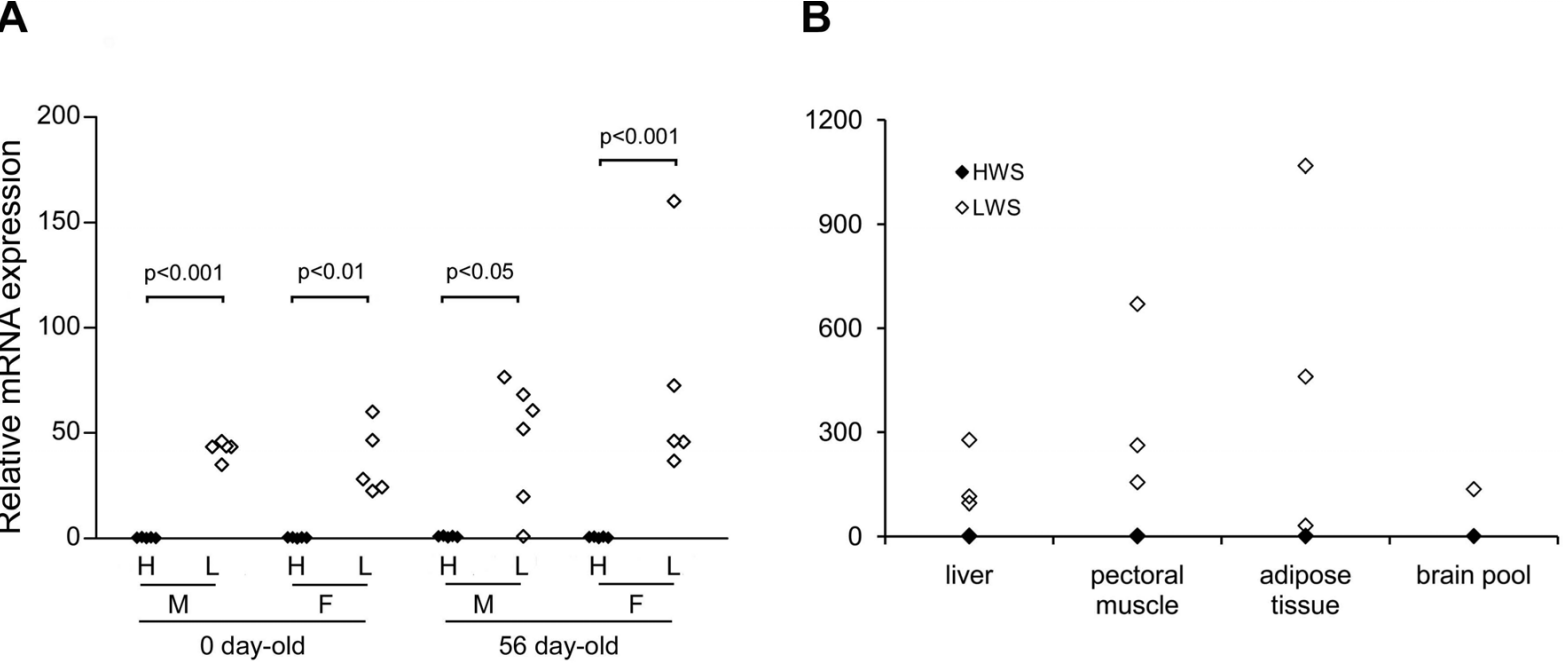
**Differential expression of ALVE in brain and peripheral tissues of HWS and LWS chickens**. Relative mRNA expression levels of *env *gene were measured using qRT-PCR with a primer pair b* shown in table 1 and figure 1B. **A**. Validation of differential expression of ALVE genes in cDNA microarray experiment. One-way ANOVA together with Newman-Keuls test as a *post-hoc *analysis was utilized. **B**. ALVE expression in peripheral tissues of HWS and LWS lines. Peripheral tissues were dissected from chickens on day 56 and the brain from chicks at hatch. N = 3 for each of HWS and LWS lines in all peripheral tissues and cDNA samples from five birds were pooled for the brain pools. H, HWS; L, LWS; M, males; F, females.

Primers for qRT-PCR were designed against the *env *gene region in the most differentially expressed sequence CN220264 (primer b*, Table 1, Fig. 1B). Five or 6 individuals from each age and sex were analyzed to confirm the differential levels. The ALVE expression in HWS chickens was notably homogenous at a very low level at both ages and for both sexes, while LWS chickens expressed high ALVE levels with individual variation (Fig. 2A).

We tested whether the high ALVE levels were specific to LWS brain tissue. Peripheral tissues from HWS and LWS 56 days-old chickens (G45) were analysed and high ALVE mRNA levels were found in all brain, liver, pectoral muscle and adipose tissues analyzed (Fig. 2B).

### Genetic transmission

**Figure 3:**
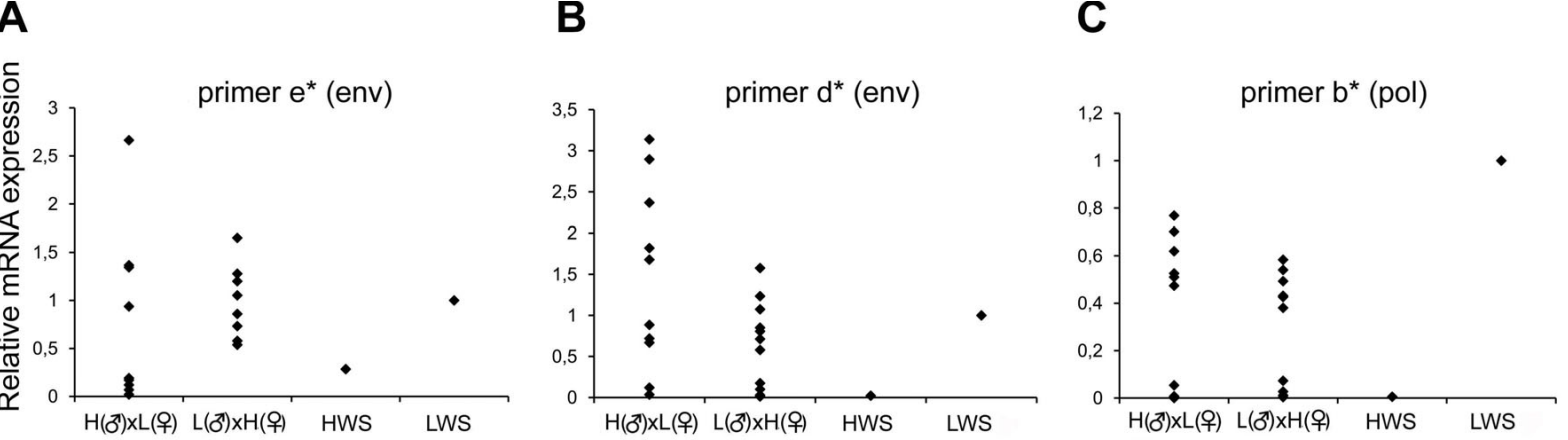
**Expression levels of ALVE genes in F_1 _birds of a reciprocal HWS × LWS crosses**. Two different pairs of primers were designed against *env *(**A **and **B**, primer pairs c* and d* in table 1 and figure 5C) and one against *pol *(**C**, primer a*) in the ALVE genome. H(♂)xL(♀) represents F_1 _birds from HWS sires and LWS dams, and F_1 _birds in L(♂)xH(♀) are from LWS sires and HWS dams. N = 10 in H(♂)xL(♀) and N = 11 in L(♂) × H(♀).

Although transmission of an endogenous retrovirus from one generation to another is generally regarded as genetic, intact transcribed ALVE provirus has been transmitted by congenital infection [25-27]. To assess if the high ALVE expression was transmitted by congenital infection or inherited, we analyzed ALVE levels in F_1 _individuals from reciprocal HWS × LWS crosses (G46). In case of congenital infection from hen to egg, high expression in F_1 _progenies should come from crosses between LWS dams and HWS sires. In case of genetic transmission of the high ALVE levels, the F_1 _individuals would have higher levels independent of whether the dams or sires were from LWS line. Furthermore, a wide range of ALVE expression levels from the low level in HWS to the high levels found in LWS individuals should be observed in the F_1 _generation. Quantitative RT-PCR was performed with primers against three different regions of the proviral ALVE transcript (Fig. 1B). We found that some F_1 _individuals from LWS dams had low levels, while their siblings from the same LWS dams had high ALVE levels. LWS sires produced progeny with high and low ALVE levels (Fig. 3). F_1 _chickens from each reciprocal crosses had a full range of expression levels (Fig. 3). The results strongly suggest that the high ALVE expression in LWS chickens are genetically determined and not transmitted by congenital infection.

### The parental lines are susceptible to ALV infection

Chickens may be susceptible or resistant to certain ALV retroviruses depending on the specific virus adherence allele they have in the Tumour Viral locus B (*TVB*) [28]. The *TVB *locus encodes a tumour necrosis factor receptor that interacts with the Env glycoprotein and is required for the viral entry into cells [29,30]. The TVB*S1 allele allows entry of ALV subgroups B, D and ALVE, while TVB*S3 permits viral entry of subgroup B and D but not E. The TVB*R allele produces truncated receptors that do not allow entry of any ALV [31,32]. Resistance to retrovirus entry could influence ALVE expression and be associated with selection for body weight. We tentatively hypothesized that the HWS line could be resistant and the LWS susceptible to ALVE. Ten HWS and 10 LWS (G41) individuals were typed for the *TVB *allele [22]. All successfully tested 19 parental individuals were positive for the TVB* S1 allele that is susceptible for ALVE infection. One sample could not be genotyped. The tentative hypothesis was rejected and it was concluded that the lines were equally susceptible.

### Number of proviral ALVE integrations

**Figure 4:**
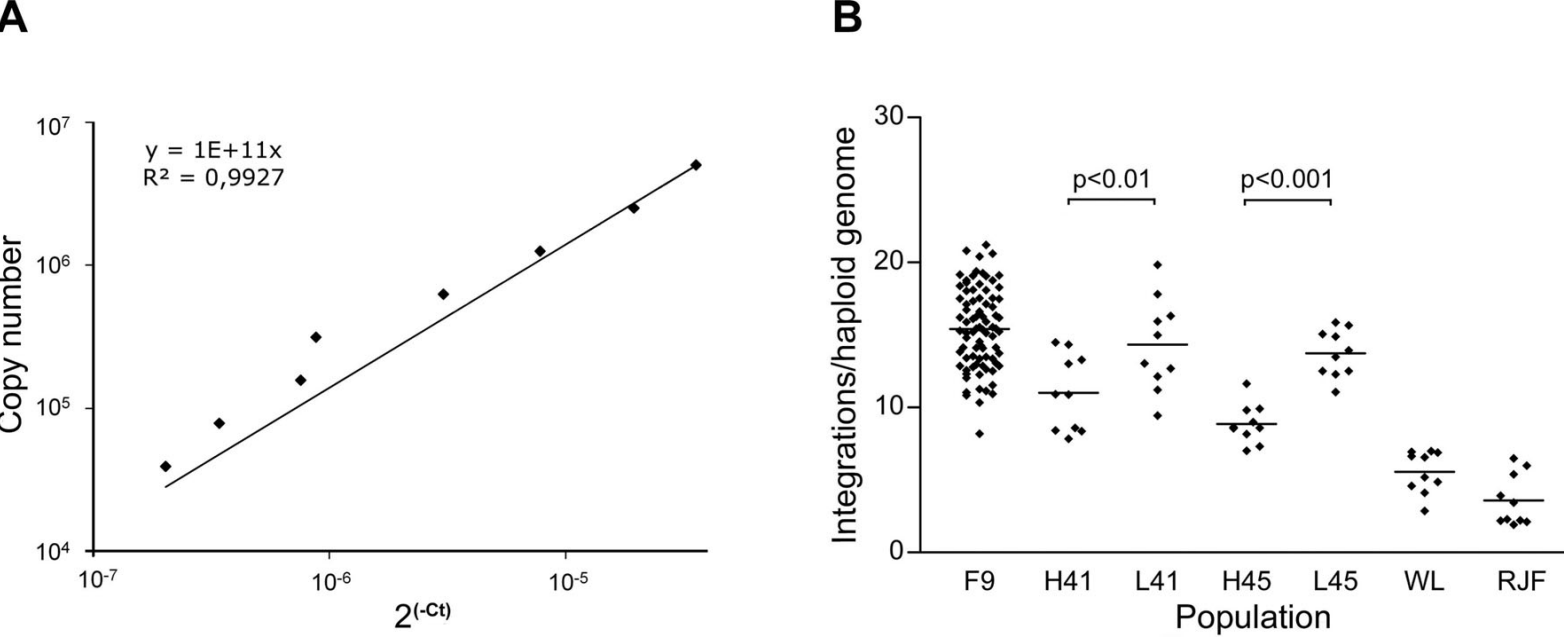
**Determination of the number of proviral integrations in different chicken populations**. **A**. Standard curve based on diluted plasmid with a PCR product. **B**. Relative copy numbers of *env *gene were examined by qPCR with primer d* and the numbers of integration per haploid genome were determined using the standard curve shown in **A**. Horizontal bars represent mean values of integration number for each population. One-way ANOVA together with Newman-Keuls test as a *post-hoc *analysis was utilized. Mean ± SEM: F9 = 15.42 ± 0.31, H41 = 11.01 ± 0.83, L41 = 14.34 ± 1.01, H45 = 8.87 ± 0.42, L45 = 13.73 ± 0.51, WL = 5.56 ± 0.46 and RJ = 3.59 ± 0.56. N = 82 in F_9 _birds and n = 10 in the other populations. F9, generation 9 of the advanced intercross line; H41 and L41, generation 41 of the HWS and LWS lines, respectively; H45 and L45, generation 45 of the parental lines; WL, White Leghorn and RJF, Red Jungle Fowl.

A central hypothesis in this work was that difference in ALVE expression levels between lines could directly contribute to the genetic differences in growth between the HWS and LWS lines. The first question was then if the number of proviral integrations differed between the lines. Therefore, we analysed the extent of proviral integration by using qPCR and by analyzing the ALVE *env *gene content in genomic DNA. In addition to the parental lines (G41 and G45), we also analysed individuals from the F_9_intercross and individuals from WL (line13) and RJF. First, a standard curve with a plasmid containing an *env *gene PCR product was made and used as an external standard for qPCR analysis (Fig. 4A). The initial result revealed that three initially selected RJF individuals had 2 to 3.5 *env *gene copies per haploid genome. This result was compared to a BLAST search of the RJF genome database (Assembly May-06) using the *env *primer sequences. Three perfect hits were found for the primer sequences; and we concluded that the qPCR analysis provided adequate results. Eighty-two F_9_, 40 from each HWS and LWS line (20 G41 and 20 G45), 10 RJFs and 10 WLHs were then analysed. The WLs and RJFs had 2 to 7 integrations, with 7 to 15 in HWS, and 9 to 20 in LWS. The difference between HWS and LWS lines was significant at both generations tested (G41 and G45). The integration numbers in individuals from each line from the two generations were not significantly different even though it was evident that G41 had a larger variance than G45 in particularly among the LWS individuals (Fig. 4B). The number of integrations in the F_9 _ranged from 8 to 22 (Fig. 4B). It is worth noting that this variance was similar in the F_9 _cross as in their parental generation (G41). A schematic outline of the parental and intercross generations is shown in Fig. 1A.

### Number of integrations in relation to ALVE expression and growth patterns

**Figure 5:**
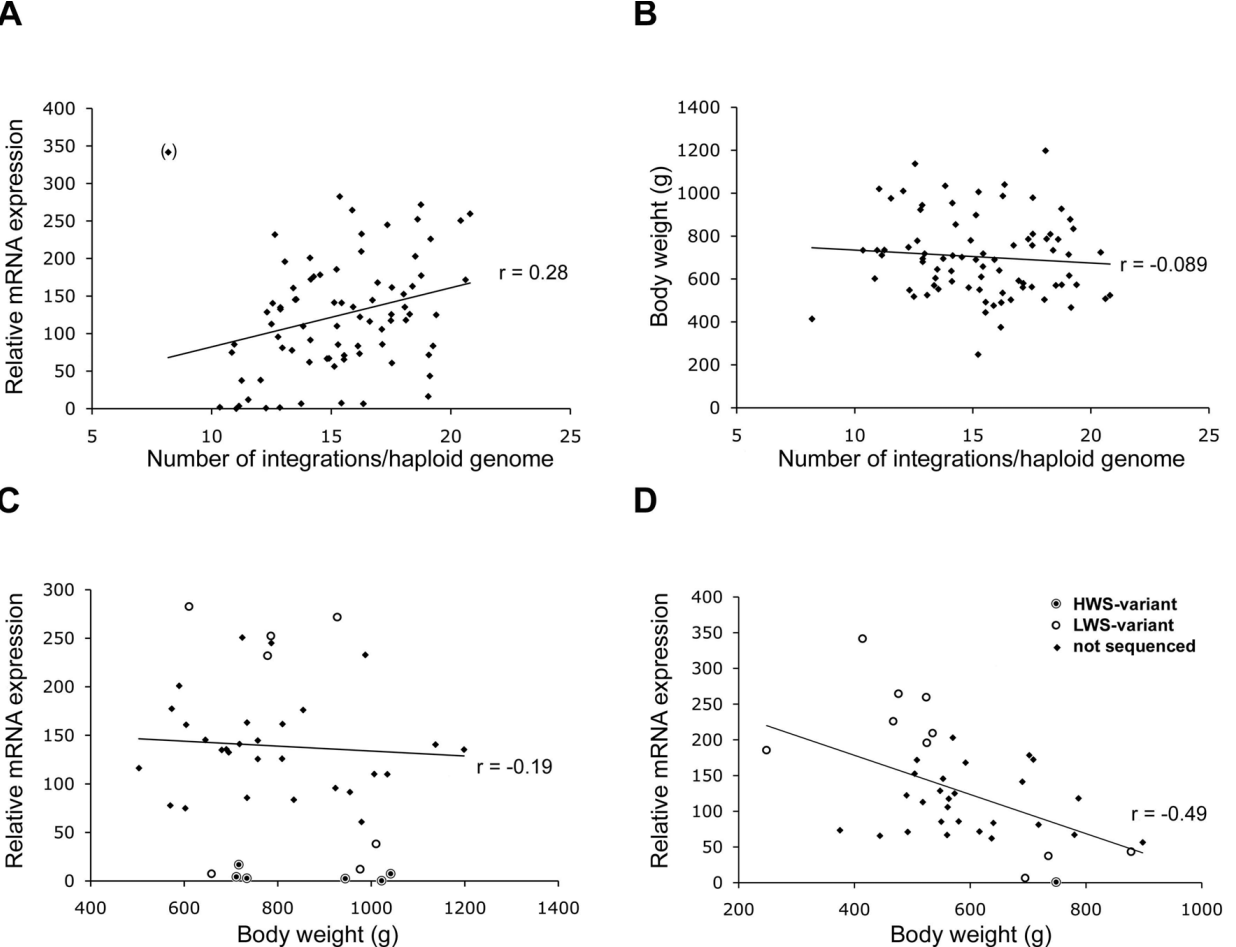
**Correlation between number-of-integrations, ALVE mRNA expression levels and body weight in F_9 _birds from the advanced intercross line**. Each dot in the plots represents an individual of F_9 _generation. **A**. Plot based on mRNA expression levels against ALVE integration number in 80 F_9 _birds. P < 0.05 for correlation coefficient r = 0.28 including all points as shown in panel A. P < 0.001 for correlation coefficient r = 0.41 when one deviating data point (black diamond) was omitted from the analysis. **B**. Plot based on body weight against the number of ALVE integration in 80 F_9 _birds. P < 0.5 for correlation coefficient. **C **and **D**. Correlation between the body weight and the ALVE expression in F_9 _birds from the advanced intercross line. Open-circled data points indicate individuals of which *env *cDNA fragments (amplicon e* in figure 1C) were sequenced in order to find out sequence variant. **C**. Plot based on 42 F_9 _male chickens. P < 0.22 for the correlation coefficient. **D**. Plot based on 38 F_9 _female chickens. P < 0.01 for the correlation coefficient. H, HWS-variant; L, LWS-variant.

The observation that the LWS chickens had more integrations and higher expression than the HWS chickens, led us to the question if the number of proviral integrations additively contributed to the different expression levels. Such association was addressed using the results from the F_9 _chickens. A correlation of 0.28 (p < 0.04) was obtained between number and expression when all F_9 _chickens were analyzed (Fig. 5A), suggesting that not all, but a few different ALVE integrations contributed to the higher expression levels in LWS.

A weak negative correlation between number of integrations and body weight for all of the F_9 _individuals was found but the trend was not statistically significant Fig. 5B. This result showed that individuals with many ALVE proviral integrations, overall did not have lower body weights (Fig. 5B) but this does not exclude the possibility that the presence of some specific integrations has a direct effect on body weight.

Next we plotted the body weight of the F_9 _individuals against the ALVE expression levels and calculated the correlation. The expression was measured with qRT-PCR using primers in the *env *gene (primer d*) with total RNA extracted from liver. The negative correlation (-0.49) between ALVE expression and body weight was highly significant for females (p < 0.01, Fig. 5D) but not for males (Fig. 5C).

### Env sequence polymorphisms in genomic and expressed sequences

We PCR-amplified and sequenced an 862 bp *env *fragment from genomic and cDNA from the two parental lines. The *env *gene is known to have the highest degree of polymorphisms in the proviral genome. The sequences obtained from genomic DNA from 21 HWS and 22 LWS individuals were polymorphic at six single base pair positions: 318, 363, 480, 749, 775 and 801 bp (Fig. 1C). The sequence result illustrated that there were fixed HWS and LWS line-specific SNPs; the HWS-variants and a LWS-variant. The HWS line had only the HWS-variants while the LWS line had all variants. The cDNA sequences revealed that the high expression levels found in LWS line constituted the LWS variant and the low expression levels in HWS individuals constituted the HWS-variant.

The *env *fragment was also amplified and sequenced from cDNA from 24 F_9 _chickens (13 males and 11 females) with high or low expression, eleven with high *env *expression and 13 with low *env *expression. The individuals are indicated in Fig. 5C and 5D. All 11 individuals with high ALVE expression and 6 individuals with lower expression had the LWS-variant of the DNA sequence. The 7 chickens with the HWS-variant were among the ones with lowest *env *expression. Six were males and only one female.

## Discussion

In this study we pursued the observation that high expression of an endogenous retrovirus of the ALVE type was associated with low growth in one of two chicken lines established by long term divergent selection for high or low body weight [5,7]. We conclude that the high levels in the LWS line show Mendelian inheritance. LWS birds have more ALVE integrations than HWS birds, which in turn have a larger number of integrations compared with WL and RJF chickens. Using F_9 _birds from an advanced intercross between the two selected lines we tested if there was a correlation between body weight, ALVE integrations and expression levels. The results indicated that a minority of the integrations contributed to the higher levels and that high expression was significantly correlated to lower body weights of females but not males. The conserved correlation between high ALVE expression and low body weight in females after 9 generations of intercrosses indicates that ALVE loci conferring high expression are genetically linked to or constitute loci directly contributing to low body weight of LWS chickens in a sex-limited fashion.

The chicken genome contains four families of ERV elements classified as chicken repeat 1 (CR1) elements, ALVE*s*, avian retrotransposones from the chicken genome (ART-CHs) and endogenous avian retrovirus elements (EAV-0) [8]. Although the microarray contained probes with different retroviral sequences, only ALVE-related sequences were identified as differentially expressed. The *env *gene in the ALVE proviral genome is a source for genetic diversity through recombination with exogenous viruses [33,34]. The sequence diversity of this gene constitutes the basis for defining the six subgroups of ALV (A, B, C, D, E, J) and is related to variation in infection susceptibility, receptor interference as well as antibody neutralization [8]. The *env *gene was used as target for the primer design for qPCR, qRT-PCR and for sequencing. The primers we used amplified the endogenous *ev*-loci of several ALVE subtypes, but did not match other types of retrovirus such as RSV or avian myeloblastosis virus. Primers against the ALVE *pol *gene confirmed the differential expression seen with the *env *primers (Fig. 3C).

Endogenous retrovirus elements are in most cases transmitted genetically [35]. Transmission of ALV can occur via several natural routes [11]. Exogenous ALVs are transmitted horizontally by infection between individuals or vertically from hen to progeny *in ovo *by congenital transmission [11,36]. Horizontal transmission is relatively inefficient while congenital transmission is very efficient and leads to a high ratio of infected embryos [8]. The ALVE elements exist in the chicken genome as partial or complete ALVE proviral genomes. Endogenous elements have in general a limited or restricted ability to transmit virus congenitally, in contrast to exogenous ALV that undergo highly efficient congenital transmission [37,38]. However, it was demonstrated that some *ev*-loci that encode complete provirus genomes, particularly *ev*-12 and *ev*-21, can be transmitted at higher frequencies from subgroup E susceptible dams to susceptible progeny [25-27].

Susceptibility of chickens to ALV retroviral infection is regulated by subtype-specific cell membrane receptors that interact with the Env glycoprotein. Exogenous ALV subtypes B and D, and virus particles of endogenous ALVE infect through this interaction. Different types of receptors for ALV subtypes B, D and E are encoded by three alleles of the *TVB *locus. The TVB*S1 allele encodes tumour necrosis factor receptors that are required for the viral entry of all three subgroups while TVB*S3 permits viral entry of subgroup B and D but not E. The TVB*R allele produces truncated receptors that do not support entry of any ALV [28,31,32]. All of the successfully tested 19 individuals (G41) possessed the TVB*S1 allele that gives susceptibility for ALVE. This result is in agreement with that 83% of chickens from 36 broiler lines were homozygous for TVB*S1 [39]. Hence, both HWS and LWS chickens are susceptible for ALVE infection and polymorphism in the *TVB *locus is neither a result of the long term selection nor is it likely to be involved in the high ALVE expressing phenotype.

The possibility that the LWS chickens propagated high ALVE expression via congenital infection from hen to egg was examined. We analyzed ALVE expression in an F_1 _generation after a reciprocal cross between the lines (G46). F_1 _siblings from the same LWS dam often had both high and low ALVE levels and LWS males transmitted high expression to their progeny (Fig. 3). Moreover, hens with high ALVE expression did not always transmit high expression to their progeny as would have been expected by congenital infection. Rather, their expression spanned the full range of expression levels seen in the parentals. Therefore, the high/low ALVE expression levels were likely to have been inherited and these data support a Mendelian mode of genetic transmission of ALVE expression. Furthermore, an exogenous ALV infection among parental LWS is less plausible because ALV-related disease symptoms have not been observed during the course of selection [5]. It cannot be excluded that such infection has occurred and by recombination may have formed elements that triggered increased ALVE expression because there are examples of male-mediated congenital transmission of ALVE [40]. The active transcription of ALVE in the tested tissues may also have introduced recombinant somatic ALVE pro-viral integrations [34].

The number of *env *gene integrations in RJF and WLs ranged from 2 to 7 per haploid genome. Both the HWS and LWS lines had more integrations than RJF and WLs. HWS individuals had significantly fewer integrations than LWS while the F_9 _birds had 8 to 22 *env *integrations per haploid genome, a number similar to that for the LWS line (Fig. 4B). The reported average for layer chickens is 1 to 3 elements, while that for meat-type chickens is 6 to 10 [15]. Altogether 22 different ALVE loci have been identified in WLs and current estimates suggest that there may be over 50 different loci [41]. Although the number of ALVE integrations in the genome pool of the White Plymouth Rock founder population for the selection experiment is not known, they probably had a similar number of *ev*-loci as the HWS and LWS lines (7 to 22 integrations). This number is little higher than the average meat bird, however, the qPCR in this study may be more sensitive than previously used methods.

HWS birds have low ALVE expression and fewer ALVE integrations than LWS birds suggesting that differential selection for growth has influenced both ALVE expression and integration number (Figs. 2 and 5). This hypothesis was supported by results from the F_9 _population where we observed a weak but significant correlation between integration number and expression (Fig. 5A). The results suggested that only a few of the integrations contributed to the high levels of expression. This assumption was further supported by the occurrence of sequence polymorphism for the *env *gene (Fig. 1C), and one sequence variant was exclusively found in LWS birds. Only this LWS-variant was found in cDNA from LWS birds and F_9 _individuals with high ALVE expression (Figs. 5C, D and 1C). In contrast, in genomic DNA from LWS chicken both the LWS- and HWS-variants were present and the HWS variant was more frequent. Thus, while LWS-variant integrations are fewer than the HWS-variant they contributed more to the high levels of expression in LWS individuals and certain F_9 _birds. An obvious interpretation is that selection for high body weight has been effective to purge or silence high expressing ALVE loci. Another possible explanation is that a previous ongoing infection would have produced novel integrations that led to the increased levels in the LWS line. For this to occur would require novel integration in the germ line in order to transmit to the next generations.

Our data from the F_9 _generation suggest that the actively expressed ALVE loci are causing reduced growth and that this effect is more pronounced in the females than in males. This pattern may be explained by a sex-specific response or because the effects by high ALVE expression are more penetrant for smaller birds and pullets are over-all smaller than males. ALVE integration is of interest for the poultry industry because the frequency of integration alters the responses to selection for economical traits [9,10,12-15]. The mechanism may be that integrations directly or indirectly disrupt other genes [8,42]. However, in humans there are only rare examples where a recessive monogenic disorder is caused by HERV integration disrupting gene function. Alternatively, a high virus expression load such as in the LWS line may affect the growth indirectly. The activation of inflammatory cytokines such as the interferon-gamma, TNF-alpha, interleukin-1 and -6, their receptors and signalling pathway components are signatures of retrovirus infection [43,44]. Such genes were not over-represented in the cDNA array analysis results [7]. Factors that regulate retrovirus trans-cellular transport and budding are also regulated at high virus loads such as actin-related modulators including Rho-like factors and trans-golgi factors [44]. Similar activation patterns have been seen after avian RSV infection of chick fibroblasts [45]. The budding of enveloped RNA viruses, including HIV and other retroviruses, usurp a cellular pathway that is normally used to form vesicles and transport them into multi-vesicular bodies [46]. Some of the differentially expressed genes observed in our previous study [7] while associated with alterations in neuronal plasticity are also regulated during acute and chronic retrovirus infections [45,47]. These include vesicle trafficking systems such as the ARL/ARF factors and FKBP5 as well as the Nephroblastoma overexpressed gene (Nov). Nov was reported to decrease in fibroblasts after Rous sarcoma virus transformation [45] and we observed lower Nov expression in LWS chicken than in HWS chickens. Nov was initially identified as a cellular gene in chick nephroblastomas induced by the retrovirus myeloblastosis-associated virus [48]. The identification of Nov as being differentially expressed between lines indicates that the expressed endogenous ALVE sequences may influence cellular gene expression and may therefore contribute to the selection response for growth.

Both HWS and LWS pullets showed delayed age of onset of egg production, and a considerable proportion of LWS females never mature [49,50]. Delayed sexual maturity for LWS females were attributed to anorexia because it was possible to induce egg laying by force-feeding. Moreover, sexually matured LWS females were heavier at 56 days of age than those that did not show sexual maturation later in life [51]. Other studies have also indicated a relationship between viral integration and traits related to reproduction. Gavora et al [10] reported that certain virus-producing *ev*-loci, ev-10 or 19 and 12, and silent gene *ev*-1 can affect egg productivity for layers. Also, the total number of *ev*-loci per genome was significantly related to body weight at first egg and mature body weight [52]. The body weight of LWS juvenile females is related to that of sexually matured LWS females and sexual maturation might be related to the number of ALVE integration and the ALVE expression. Therefore, it is not surprising that high expression of ALVE is correlated to the low juvenile body weight in female chickens.

Quantitative trait locus (QTL) analysis has been performed after crossing the HWS and LWS lines and more than 13 growth-related QTLs were identified all with minor individual effects [16,53] and a high degree of epistasis [54]. Although the exact location of ALVE integrations remain to be defined, our results are consistent with the QTL data in that we present data that multiple proviral loci together contribute to one aspect of the phenotype, namely to the low weight of pullets.

## Conclusion

Artificial selection for high or low juvenile body weight was associated with high frequency and elevated expression levels of ALVE loci in the LWS line. Although the genomic location remains ambiguous, it is most likely that ALVE loci were genetically inherited from both HWS and LWS chickens. Analysis of the advanced intercross line demonstrated significant correlation between low body weight and high ALVE expression. The results showed that while LWS chickens have accumulated more ALVE integrations than HWS ones, only a few of the integrations contribute to the high expression levels observed in the LWS line. High ALVE expression among F_9 _birds was significantly correlated with low body weight for the females but not for males. The conserved correlation between high expression and low body weight in females after 9 generations of intercrosses, indicated that ALVE loci conferring high expression are genetically linked to or constitute in part the loci for a low body weight of the pullets.

## Competing interests

The authors declare that they have no competing interests.

## Authors' contributions

SKa did all experimental work and contributed to the writing of the manuscript. SKe contributed to PCR amplifications, TVB typing as well as the sequence analysis. LB and UL did the integration analysis. PBS produced the chicken lines and conceived the project together with LA and FH. FH and LA supervised the work and FH wrote the manuscript. All authors read and approved its final version.
